# Age-Related Differences in miRNA Expression in Mexican-American Newborns and Children

**DOI:** 10.3390/ijerph16040524

**Published:** 2019-02-13

**Authors:** Karen Huen, Daneida Lizarraga, Katherine Kogut, Brenda Eskenazi, Nina Holland

**Affiliations:** 1 Children’s Environmental Health Laboratory, Global Berkeley Campus, University of California, Berkeley, 1301 S. 46th Street, Bldg 112, Richmond, CA 94804, USA; daneida.lizarraga@gmail.com (D.L.); ninah@berkeley.edu (N.H.); 2 Center for Children’s Environmental Health, School of Public Health, University of California, Berkeley, 1995 University Avenue Suite 265, Berkeley, CA 94720, USA; kkogut8@berkeley.edu (K.K.); eskenazi@berkeley.edu (B.E.)

**Keywords:** miRNA, blood, age

## Abstract

Epigenetic mechanisms have emerged as an important pathway through which environmental exposures can affect health through the regulation of gene expression without changes in DNA sequence: microRNAs (miRNAs) are short non-coding RNAs that target protein-coding mRNAs, leading to post-transcriptional repression. They are involved in important physiologic processes, but little is known about how miRNA expression may change with age in children. We used an nCounter miRNA assay to assess the expression of 43 miRNAs in buffy coat samples collected from newborns (*n* = 121) and 7-year-old (*n* = 142) children. We identified 36 miRNAs that were differentially expressed between newborns and 7-year-olds after controlling for blood cell composition. Using pathway analysis, we found that differentially expressed miRNAs targeted genes enriched for processes related to post-translational modifications, metabolism, and immune response. Our study found that unlike adults, where miRNA expression levels in peripheral blood may decrease with age, expression levels of most miRNAs increased from birth to mid-childhood. This may be reflective of the role miRNAs may play in the highly coordinated mechanisms regulating genes involved in children’s development. Furthermore, it will be important to adjust for both age and blood cell composition in future pediatric studies of miRNA expression in blood.

## 1. Introduction

Epigenetics has emerged as an important biological mechanism through which environmental exposures can affect health [1,2,3]. Epigenetic marks such as DNA methylation, non-coding RNA, and histone modifications regulate gene expression without changes in DNA sequence. The majority of human studies have focused on the role of DNA methylation on gene regulation, demonstrating associations of aberrant methylation profiles with environmental exposures such as smoking, phthalates, and arsenic [4,5], as well as adverse health outcomes ranging from low birthweight to cancer [6,7].

Growing evidence has demonstrated that non-coding RNAs also play a major role in important cellular processes and disease etiology [8]: miRNAs are short non-coding RNAs of about 18–22 nucleotides in length. They bind to specific mRNA sequences via miRNA binding sites, thereby blocking translation. Like DNA methylation, several studies have reported associations of environmental exposures, including phthalates and air pollution, with differences in miRNA expression [9,10]. Furthermore, miRNAs have been associated with health outcomes such as fetal growth [11], cancer [12], and cardiovascular disease [13].

Host factors including sex and age can also influence interindividual differences in epigenetic profiles. Yet little is known about sex- and age-specific differences in human miRNA expression, particularly in children. In adult human plasma, 7 out of 179 candidate circulating miRNAs have been associated with sex [14]. We recently published the first study comparing the miRNAome between newborn boys and girls (*n* = 89) and found 94 miRNAs differentially expressed by sex in cord blood: The mRNA targets of these differentially expressed miRNAs were significantly enriched in pathways related to neurodevelopment, RNA metabolism, and post-transcriptional gene silencing [15].

Age-related changes in miRNA expression may be one mechanism regulating gene expression at different developmental stages. Only a few studies have examined age-associated differences in miRNA expression in children. Over 100 liver miRNAs (out of 533) were differentially expressed among fetal, pediatric, and adult subjects, with the largest differences observed between fetal and pediatric liver samples [16]. Among adults, the majority of miRNAs expressed in peripheral blood have exhibited lower expression levels in older people [17].

While studies on the relationship between age and sex and miRNA expression are still limited, the existing data suggest that consideration of important host factors is critical for epidemiological studies of miRNA expression. In the present study, we sought to better understand age-related differences in miRNA expression among newborns and 7-year-old children.

## 2. Materials and Methods

### 2.1. Study Subjects

Children and mothers in this study were participants of the Center for Health Assessment of Mothers and Children of Salinas (CHAMACOS) birth cohort, a longitudinal study of farmworker families from the agricultural region of Salinas Valley, California [18]. Pregnant women were enrolled in the study from 1999 to 2000. Women were eligible to enroll if they were at least 18 years of age, at fewer than 20 weeks’ gestation, Spanish- or English-speaking, eligible for low-income health insurance, receiving prenatal care at one of the participating community clinics, and planning to deliver at the local public hospital. This study included 196 randomly selected children with cord blood (*n* = 121) and/or a blood sample from age 7 years (*n* = 147) available for analysis: 79 children had samples assessed at both time points. Children included in this study did not differ significantly from all children in the cohort by demographic and exposure variables (e.g., poverty level, sex, maternal marital status, maternal prenatal farm work status, or maternal use of alcohol or tobacco during pregnancy). Furthermore, children with measurements available at both ages compared to those with measurements at only one time point did not differ by demographic and exposure variables, with the exception of being overweight at age 7 years. There was a suggestive trend of a higher incidence of being overweight and obesity among the children with only a single time point (*p* = 0.05), but this association did not remain statistically significant after adjustment for multiple testing.

Study protocols were approved by the University of California, Berkeley, Committee for the Protection of Human Subjects (2010-03-949). Written informed consent was obtained from all mothers, and children provided verbal assent at age 7.

### 2.2. Blood Collection and Processing

Blood specimens were collected from CHAMACOS children at birth (umbilical cord blood) and at approximately 7 years of age (by venipuncture). We piloted the protocol for cord blood collection in CHAMACOS participants using two assays, fluorescent in situ hybridization (FISH) analysis for sex chromosomes and polymerase chain reaction (PCR) analysis using human leukocyte antigen (HLA) markers, to assure that cord blood samples were not contaminated by maternal blood. Of the 30 samples randomly selected from CHAMACOS boys, none of the assays indicated any contamination with maternal blood. Heparinized whole blood, collected in BD vacutainers^®^ (Becton, Dickinson and Company, Franklin Lakes, NJ, USA), were centrifuged, separated into aliquots of plasma, buffy coats, and red blood cells, and then stored at −80 °C at the School of Public Health Biorepository, University of California, Berkeley.

### 2.3. RNA Isolation

In our pilot studies, we compared RNA yields from clot, serum, and buffy coat fractions (containing all white blood cells) of blood and found that the highest RNA yields came from the buffy coat fractions. Based on these data and previously published research utilizing miRNA expression data from buffy coat fractions in human populations [19,20,21,22,23], we chose to use RNA from buffy coats to analyze miRNA expression in this study. Total RNA was purified from 20 μL of buffy coat using a mirVana miRNA isolation kit (Life Technologies, Waltham, MA, USA). Concentration and quality of RNA were measured using a NanoDrop 2000 Spectrophotometer (Thermo Scientific, Waltham, MA, USA). All samples included in this analysis had a 260/280 ratio >1.9, and their average RNA integrity number (RIN) value, as measured by Bioanalyzer (Agilent Technologies, Santa Clara, CA, USA), was 8.1. Purified RNA was stored at −80 °C until analysis.

### 2.4. nCounter miRNA Expression Assay

In our previous study, we used an miRNAome wide approach, identifying >500 miRNAs with measurable expression in cord blood buffy coat samples using an EdgeSeq miRNA whole transcriptome assay [15]. Here, we measured miRNA expression in a set of 43 miRNAs that had measurable miRNA expression in our previous study [15] with a range of low to high expression. To determine miRNA expression, we used a custom-designed nCounter miRNA Expression Assay (NanoString Technologies, Seattle, WA, USA). We utilized 100 ng per sample of isolated total RNA for the expression assay, which was performed per the manufacturer’s recommended protocol.

### 2.5. Cell Composition

Cell type proportions were calculated in the R-package *minfi* [24] based on their methylation signatures in Illumina 450K data that were previously analyzed [25,26]. For cord blood samples, we estimated cell-type proportions using a recently published cord blood reference dataset that included nucleated red blood cells [27]. For blood samples collected from 7-year-old-children, we used an adult reference dataset [28].

### 2.6. Statistical Analysis

Raw miRNA counts measured by nCounter were imported for quality control and normalization using proprietary software from NanoString, nSolver 3.0 (NanoString Technologies, Seattle, WA, USA). Raw miRNA expression counts were normalized using the geometric mean of all miRNAs with mean raw counts >50. To determine whether variability of miRNA expression increases as miRNA expression increases, we calculated the Pearson’s correlation coefficient of log_2_ mean miRNA expression with log_2_ mean SD of miRNA expression at each time point. To model relationships of miRNA expression with white blood cell counts, we constructed separate linear regression models for each miRNA (outcome) at each time point (birth and age 7 years), including blood cell type proportions as the independent variables in the model. To determine whether the miRNAs were differentially expressed by sex at each age, we used the R/Bioconductor package *limma*, controlling for white blood cell composition.

To determine correlations of miRNAs across time points, we also calculated the partial correlation of each miRNA at birth and age 7 years, controlling for cell composition, among children with miRNAs assessed at both ages (*n* = 79). All other analyses included all children (121 newborns and 142 7-year-olds). To determine whether children’s miRNA expression levels cluster by age, we applied principal components analysis (PCA) to log_2_ normalized miRNA expression counts using the *princomp* command in R. We also used the *pca3d* package in R to visualize the results by age group. To determine which miRNAs were differentially expressed between the two time points, while controlling for blood cell composition, we also utilized the *limma* package.

To determine the potential regulatory function of miRNAs differentially expressed between newborns and 7-year-old children, we used DIANA miRpath v3.0, a web-based computational tool [29] to conduct pathway analysis. This tool carries out target prediction of miRNAs using a microT-CDS algorithm followed by pathway enrichment analysis of the miRNA target genes using gene ontology (GO) pathways. The false discovery rate (FDR)-adjusted *p*-value threshold was set to 0.05, and the microT threshold was set to 0.8. Reduce + visualize gene ontologies (REVIGOs) were used to visualize and categorize GO terms via semantic similarity dimension reduction [30]. Allowed similarity was set to medium (0.7) using the *Homo sapiens* GO term database.

Unadjusted *p*-values <0.05 were considered significant. However, to account for multiple testing, we also used the Benjamini–Hochberg (BH) method for false discovery rate (FDR) with an FDR *q*-value < 0.05 threshold for significance [31]. Statistical analyses were performed in STATA (version 12.0, StataCorp, College Station, TX, USA) and R (version 3.4.3, R Foundation for Statistical Computing, Vienna, Austria).

## 3. Results

### 3.1. Characteristics of Study Participants

Table 1 describes characteristics of CHAMACOS children included in this study at birth and age 7. There were slightly more girls (58% and 55%) than boys (42% and 45%) at each age, respectively. The majority of children were normal weight at birth. By age 7, over 50% of children were either overweight or obese.

### 3.2. miRNA Expression in Newborns and 7-Year-Old Children

**Figure 1:**
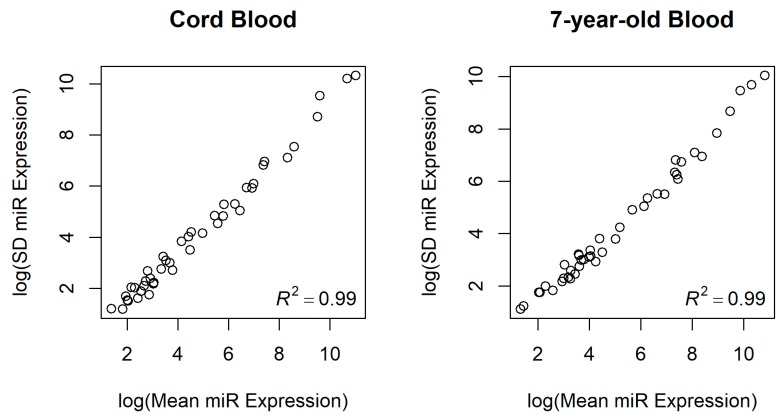
Scatter plot of mean miRNA expression levels and standard deviation of miRNA expression levels in newborns and 7-year-old children. Log mean miRNA expression levels and log standard deviation of miRNA expression levels were highly correlated among the 43 miRNAs interrogated by NanoString. SD: standard deviation.

We measured miRNA expression in a set of 43 miRNAs that had measurable miRNA expression in our previous study [15] with a range of low to high expression. Distributions of normalized miRNA counts in newborns (cord blood) and 7-year-old children are summarized in Table 2. Mean expression counts of the 43 miRNAs ranged from 1.3 to over 60,000 in newborns and from 1.0 to almost 50,000 in 7-year-old children, representing a range of low-, medium-, and high-expressing miRNAs in blood cells. Additionally, standard deviations of miRNA expression were very strongly correlated with mean miRNA expression levels (Figure 1), demonstrating that, as expected, more highly expressed miRNAs in blood cells also tended to be more variable.

### 3.3. miRNA Expression and Blood Cell Count Proportions

Blood cell proportions estimated by *minfi* in newborns and 7-year-old children are shown in Table S1. As expected, cord blood contained a measurable amount of nucleated red blood cells, unlike 7-year-old blood. Table 3 summarizes miRNAs that had suggestive associations (unadjusted *p*-value < 0.05) with blood cell proportions at either age. Among the 43 miRNAs assessed, we found 8 miRNAs that had suggestive associations (2 positive and 6 negative), with blood cell proportions in cord blood (Table 3, Table S2) indicating that white blood cell proportions may influence expression of some miRNAs. In addition, miR-495-3p, the only miRNA with a suggestive association with blood cell proportions in 7-year-old children (Table 3, Table S3), was also associated with CD4+ T cell and monocyte proportion in cord blood, indicating a consistency of this relationship at both ages. However, none of the associations of miRNA expression with blood cell composition remained statistically significant after controlling for multiple testing (FDR-adjusted *p*-values > 0.05).

### 3.4. Differential miRNA Expression by Sex in Newborns and 7-Year-Old Children

To examine whether the 43 miRNAs assessed in cord and 7-year-old blood samples differed by sex, we performed differential expression (DE) analysis for sex. We observed a very consistent trend of lower expression in girls compared to boys among the majority of cord blood miRNAs (32 out of 43), as indicated by the negative fold-change values shown in Table S4. Among 7-year-olds, roughly half (22 of 43) of the miRNAs had lower expression levels in girls. We found 14 miRNAs (including let-7d-5p and miR-301-3p) that were lower in girls compared to boys at both ages and 3 miRNAs (miR1254, miR-139-5p, and miR-4461) that were higher in girls compared to boys at both ages. However, none of the differences by sex were statistically significant after controlling for the FDR (FDR-adjusted *p*-values were all >0.05).

### 3.5. Differential miRNA Expression by Age

For each miRNA, we calculated the partial correlations between newborn and 7-year-old expression, controlling for cell composition among the 79 children with measurements at both ages (Table S5). Correlations (rho) were weak to moderate at best, with absolute values ranging from 0.01 to 0.23. None of the correlations were statistically significant, suggesting that miRNA expression was not correlated between newborns and 7-year-old children.

**Figure 2:**
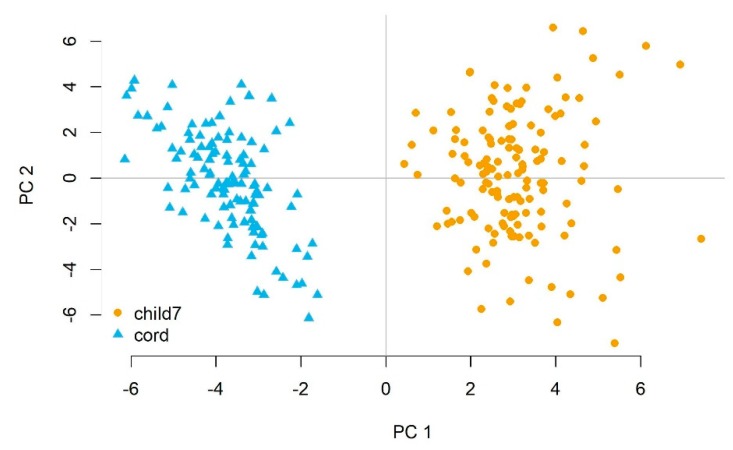
Principal components analysis (PCA) plots of miRNA expression in newborns and 7-year-old children. PCA analysis showed a clear separation of miRNA expression between age groups along the first principal component.

We performed principal components analysis on all miRNAs assessed in newborns and 7-year-old children. The first four principal components accounted for >60% of the variability of miRNA expression levels (Figure S1). Figure 2 shows the PCA plot of the first two principal components, accounting for 29% and 14% of the variance, respectively. We found that there was distinct separation between newborns and 7-year-old children along the first principal component, demonstrating that age accounted for the largest portion of the variability of the miRNAs assessed.

**Figure 3:**
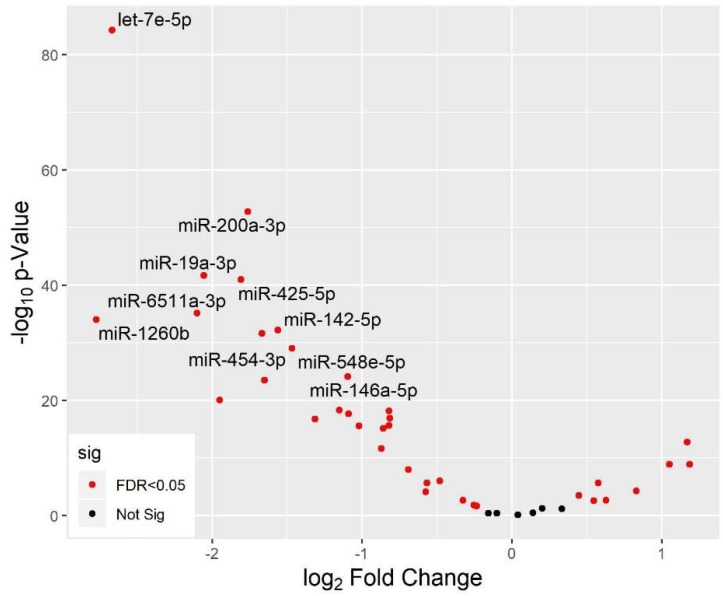
Volcano plots of differential miRNA expression between newborns and 7-year-old children. The majority of miRNAs had negative log_2_ fold-change values, showing lower expression in newborns compared to 7-year-old children.

**Figure 4:**
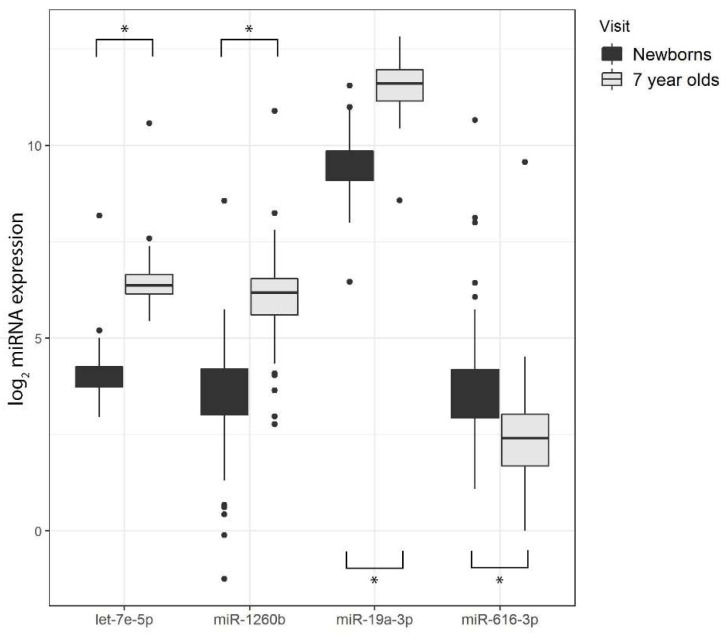
Boxplots of 4 of the 36 miRNAs differentially expressed between visits. As an example, distributions of four miRNAs that were differentially expressed between newborns and 7-year-olds are shown here. The first three box plots show higher miRNA expression in 7-year-old children for let-7e-5p, miR-1260b, and miR-19a-3p, while miRNA expression was actually higher in newborns for miR-616-3p. Asterisks (*) indicate statistically significant associations (FDR-adjusted *p*-values < 0.05).

In addition to PCA, we also used the R/Bioconductor package *limma* to identify miRNAs differentially expressed between newborns and 7-year-old children, adjusting for white blood cell proportions. Table 4 and Figure 3 summarize our results. The majority of miRNAs (28 out of 43 miRNAs) were lower in newborns compared to 7-year-olds, as demonstrated by negative fold-change values that were highly statistically significant after controlling for the FDR (FDR-adjusted *p*-values < 0.05). For example, miR-1260b was 6.8-fold lower in newborns compared to 7-year-old children (FDR-adjusted *p*-value = 7.29 × 10^−34^). There were also a few miRNAs (8 out of 43) with positive fold-change values, indicating a small numbers of miRNAs with higher expression in newborns compared to 7-year-old children. Figure 4 shows distributions of 4 of the 36 miRNAs that were differentially expressed by age. It includes several that followed the overall trend of lower levels in newborns than in 7-year-olds (e.g., miR-19a-3p, miR-1254, miR-425-5p, miR-1260b, etc.). However, other miRNAs did not follow this pattern. For instance, expression levels of miR-616-3p, which was also differentially expressed between the two age groups, were higher in newborns versus 7-year-old children.

### 3.6. Pathway Analysis of miRNA Differentially Expressed between Ages

**Figure 5:**
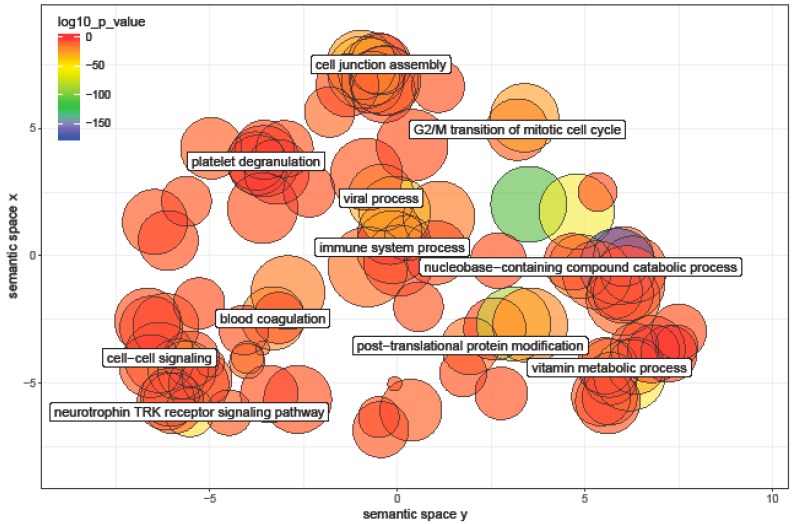
Enriched gene ontology (GO) terms: miRpath v3.0 was used to identify significantly enriched GO terms among predicted mRNA targets of 36 miRNAs that were differentially expressed between newborns and 7-year-old children. The miRNA-related GO terms related to age differences in children fell into several broad categories including immune system process, neurotrophin tropomyosin-related kinase (TRK) receptor signaling, post-translational protein modification, and mitotic cell cycle.

We applied DIANA-miRPath in miRNAs differentially expressed between newborns and 7-year-old children. Using the union of predicted genes targeted by differentially expressed miRNAs, we found 130 gene ontology (GO) terms that were significantly enriched (FDR-adjusted *p*-value < 0.05, Table S6). The most significant GO terms included metabolic processes, ion binding, and cellular protein modification processes. Using REVIGO to summarize and visualize the GO terms, we found that the most prominent miRNA-related GO terms related to differences between newborns and 7-year-old children fell into several broad categories such as cell cycle, cell–cell signaling, immune system process, post-translational modification, and catabolic and metabolic processes (Figure 5).

## 4. Discussion

In this study, we assessed miRNA expression of 43 miRNAs in buffy coat samples collected from children at birth and age 7 years. We found suggestive, though nonsignificant, evidence that expression of some miRNAs can differ by cell type, highlighting the need to consider cell type composition in studies of miRNA expression in blood. Importantly, we found several miRNAs that were differentially expressed between the two ages, the majority of which were more highly expressed in 7-year-old children compared to newborns after controlling for white blood cell composition.

Few studies have examined associations of miRNA expression with age in children. Burgess et al. identified 114 upregulated and 72 downregulated miRNAs when comparing fetal to pediatric liver samples [16]. In our study, five of the differentially expressed miRNAs overlapped with their findings, suggesting some similarities despite the different target tissues studied. Among adults, a few studies have reported age-related decreases in miRNA expression in blood [14,32,33]. The most comprehensive study to date measured 150 miRNAs in whole blood samples from over 5000 adults [33]. They found 127 miRNAs that were differentially expressed in relation to chronological age. Notably, many of the miRNAs differentially expressed between newborns and 7-year-old children in our study (16 of 43) were also differentially expressed by age in adults, though not necessarily in the same direction. It is possible that some miRNAs remain involved in age-related biological pathways throughout the life course, while others may only be implicated in developmental pathways in children or in aging and senescence in adults.

In children, miRNAs related to age may be involved in biological pathways related to development and growth. Our pathway analysis highlighted several broad categories of GO terms that were significantly enriched among age-associated miRNAs, including immune system process, post-translational modification, and cell cycle. Among adults, age-associated miRNAs were also enriched for pathways related to immune response and the regulation of transcription and translation [33]. These data suggest that these miRNAs may regulate genes that are integral to age-related biological mechanisms.

It is well established that DNA methylation levels vary between blood cell types in humans [34], and it is now standard practice to control for blood cell proportions in DNA methylation studies [35,36]. However, how miRNA expression levels differ by blood cell composition is not as well characterized. One recent study examined miRNA expression in seven types of peripheral blood cells and identified several blood cell-specific miRNAs, indicating that miRNA expression profiles can indeed differ between blood cell types [37]. In our study, we found suggestive but nonsignificant associations of some miRNAs with blood cell type proportions. This corroborates the Huan et al. finding [33] that although blood cell composition does account for some variability in miRNA expression levels among adults, adjusting for blood cell composition in statistical models does not considerably change results.

Sex-specific differences in miRNA expression have been previously reported in a few studies [14,38], though data in children are quite limited. None of the 43 miRNAs assessed in this study were significantly differentially expressed between boys and girls at either age. These results corroborate both our previous study of 89 newborns [15] and another study of cord blood miRNAs [39]. Interestingly, we did observe some consistent, albeit nonsignificant, trends of lower miRNA expression in girls compared to boys, particularly in newborns, which also confirms trends observed in our previous study.

Our study was performed in buffy coat samples that contained a mixture of different white blood cell types, which had the potential to bias the results if miRNA expression differed between these various blood cell types [37,40]. However, adjustment for blood cell proportions did not significantly change results, and we found only weak evidence of relationships between miRNA expression levels and blood cell type proportions. Furthermore, samples were stored at −80 ℃ without RNA stabilization, which may have presented another potential source of bias for miRNA expression levels. However, miRNAs are considered to be relatively stable compared to other RNA molecules [41]. Another limitation of this study was that we only examined a subset of miRNAs expressed in blood. Given that many miRNAs were differentially expressed by age, it will be important to expand analysis to all miRNAs with measurable expression in blood. Despite these limitations, this is one of the few studies that has examined potential differences in miRNAs by age in children and has identified miRNAs that may be essential as children develop from infancy to mid-childhood.

## 5. Conclusions

In this study, we observed differential expression of many miRNAs in newborns compared to 7-year-old children. Pathway analysis revealed several ontology terms related to post-translational modifications, metabolism, and immune response that were enriched among predicted targets of age-associated miRNAs. Given that the majority of differentially expressed miRNAs were upregulated at age seven (versus newborns), the predicted mRNA targets and their associated pathways highlighted processes important in biological development as children get older.

## Figures and Tables

**Table 1:** Characteristics of Center for Health Assessment of Mothers and Children of Salinas (CHAMACOS) children included in this study.

Child Characteristics	*n* (%)	Mean (SD)
**Newborns**		
Sex		
Boy	51 (42)	
Girl	70 (58)	
Gestational Age (weeks)		38.7 (1.5)
Birthweight		
Low (≤2500 g)	3 (2)	
Normal (>2500 g)	118 (98)	
**7-year-olds**		
Sex		
Boy	64 (45)	
Girl	78 (55)	
Age at 7-year assessment (years)		7.1 (0.3)
Obesity Status at age 7 years		
Normal (≤85th percentile)	67 (47)	
Overweight (>85th, <95th percentile)	27 (19)	
Obese (≥95th percentile)	48 (34)	

SD: standard deviation.

**Table 2:** Distributions of microRNA (miRNA) expression (counts) in newborns and 7-year-old children.

miRNA Name	MIMAT ID	Newborns			7-Year-Olds			Level of Expression
		Mean	SD	IQR	Mean	SD	IQR	
hsa-miR-223-3p	MIMAT0000280	60218.9	31065.5	34513.5	49912.1	23174.2	33122.0	High Expression (mean > 500) *
hsa-miR-150-5p	MIMAT0000451	42279.6	27052.2	28918.5	29763.7	16302.7	17273.0	
hsa-miR-25-3p	MIMAT0000081	14566.5	13796.9	8073.7	19238.3	13050.8	12311.9	
hsa-let-7b-5p	MIMAT0000063	13089.6	6215.4	6115.7	12935.8	5898.9	6897.7	
hsa-miR-26b-5p	MIMAT0000083	5192.5	1967.3	2439.9	7633.9	2618.9	3315.9	
hsa-let-7d-5p	MIMAT0000065	4024.1	1316.9	1234.8	4313.4	1095.8	1406.0	
hsa-miR-185-5p	MIMAT0000455	1640.3	1000.4	688.2	1531.7	609.6	633.6	
hsa-miR-92a-3p	MIMAT0000092	1623.7	1045.0	1031.2	1534.0	913.7	970.0	
hsa-miR-26a-5p	MIMAT0000082	1108.7	513.9	412.6	1977.9	931.9	694.0	
hsa-miR-130a-3p	MIMAT0000425	1057.6	628.1	278.4	1695.1	441.6	448.8	
hsa-miR-19a-3p	MIMAT0000073	808.2	383.2	378.4	3263.7	1234.5	1729.3	
hsa-miR-107	MIMAT0000104	628.2	159.8	175.3	1018.3	249.5	276.9	
hsa-miR-425-5p	MIMAT0003393	509.1	202.4	235.4	1609.7	527.8	645.9	
hsa-miR-199a-5p	MIMAT0000231	336.1	197.1	189.1	286.1	135.9	164.9	Medium Expression (50 < mean < 500) *
hsa-let-7c-5p	MIMAT0000064	335.3	179.6	142.9	536.8	277.9	236.1	
hsa-miR-146a-5p	MIMAT0000449	259.5	250.7	100.6	474.6	292.9	188.4	
hsa-miR-454-3p	MIMAT0003885	254.3	95.9	115.4	754.8	250.3	293.7	
hsa-miR-301a-3p	MIMAT0000688	145.0	64.6	59.0	176.8	71.0	65.3	
hsa-miR-495-3p	MIMAT0002817	131.9	376.5	64.4	45.6	126.9	28.0	
hsa-miR-136-5p	MIMAT0000448	114.9	389.5	49.5	45.1	127.1	27.7	
hsa-miR-199b-5p	MIMAT0000263	95.1	73.8	69.8	57.5	39.0	35.1	
hsa-miR-664a-3p	MIMAT0005949	89.2	35.4	47.0	151.1	47.5	63.4	
hsa-miR-130b-3p	MIMAT0000691	54.5	104.2	15.2	81.4	155.0	21.9	
hsa-miR-1289	MIMAT0005879	52.2	128.7	21.0	53.0	125.7	20.9	
hsa-miR-141-3p	MIMAT0000432	52.0	333.6	10.4	94.6	699.8	20.4	
hsa-miR-505-3p	MIMAT0002876	46.2	94.8	17.1	28.3	62.6	10.4	Low Expression (mean < 50) *
hsa-miR-4461	MIMAT0018983	41.2	217.2	9.9	46.1	253.6	11.9	
hsa-miR-377-3p	MIMAT0000730	37.5	61.7	26.1	22.2	34.1	18.2	
hsa-miR-616-3p	MIMAT0004805	32.9	149.5	10.5	11.9	63.5	4.9	
hsa-miR-335-5p	MIMAT0000765	29.5	22.9	14.8	54.9	35.0	25.0	
hsa-miR-142-5p	MIMAT0000433	29.3	85.9	9.6	74.2	220.0	22.5	
hsa-miR-139-5p	MIMAT0000250	26.9	174.8	6.1	17.6	127.3	5.6	
hsa-let-7e-5p	MIMAT0000066	18.7	25.3	5.8	98.1	123.1	30.4	
hsa-miR-766-3p	MIMAT0003888	18.5	49.2	9.5	49.0	126.0	23.8	
hsa-miR-371b-5p	MIMAT0019892	17.7	94.7	5.5	5.3	31.8	1.8	
hsa-miR-1260b	MIMAT0015041	17.7	34.3	10.4	92.1	159.3	46.6	
hsa-miR-1275	MIMAT0005929	17.4	47.9	7.4	23.2	62.9	11.1	
hsa-miR-6511a-3p	MIMAT0025479	16.3	94.7	3.9	54.6	349.4	14.9	
hsa-miR-92b-3p	MIMAT0003218	12.8	67.4	5.7	5.8	31.8	3.1	
hsa-miR-1254	MIMAT0005905	11.5	84.0	2.3	30.2	254.9	7.0	
hsa-miR-200a-3p	MIMAT0000682	11.1	10.4	3.6	32.6	31.3	11.5	
hsa-miR-548e-5p	MIMAT0026736	10.5	34.4	4.2	26.8	94.7	10.8	
hsa-miR-103a-3p	MIMAT0000101	6.7	14.8	2.8	14.7	31.4	6.7	

* Low, medium, and high expression determined by mean normalized counts of miRNA expression in cord blood. IQR: Interquartile range. MIMAT: accession number of mature miRNAs in the miRBase database.

**Table 3:** Associations of blood cell composition with newborn and 7-year-old miRNA expression.

miRNA Name	cd8+T		cd4+T		NK Cells		B Cells		Monocytes		Granulocytes		nRBCs	
	β (95% CI)	*p*-Value	β (95% CI)	*p*-Value	β (95% CI)	*p*-Value	β (95% CI)	*p*-Value	β (95% CI)	*p*-Value	β (95% CI)	*p*-Value	β (95% CI)	*p*-Value
**Newborns**														
miR-495-3p	12.38 (−4.25, 29.01)	0.143	16.39 (1.33, 31.46)	0.033	14.13 (−3.48, 31.75)	0.115	13.10 (−2.47, 28.67)	0.098	19.66 (0.28, 39.04)	0.047	13.21 (−2.29, 28.71)	0.094	14.62 (−1.07, 30.31)	0.068
miR-377-3p	16.37 (−0.78, 33.52)	0.061	20.27 (4.74, 35.80)	0.011	12.37 (−5.80, 30.53)	0.180	19.31 (3.25, 35.36)	0.019	22.68 (2.69, 42.66)	0.027	17.09 (1.10, 33.07)	0.036	19.19 (3.01, 35.37)	0.021
miR-301a-3p	6.22 (−1.73, 14.17)	0.124	9.01 (1.81, 16.21)	0.015	8.77 (0.35, 17.19)	0.041	6.21 (−1.23, 13.65)	0.101	8.8 (−0.46, 18.07)	0.062	8.27 (0.86, 15.68)	0.029	9.07 (1.57, 16.57)	0.018
miR-26b-5p	−6.64 (−15.92, 2.63)	0.159	−8.92 (−17.32, −0.51)	0.038	−5.3 (−15.12, 4.53)	0.288	−9.01 (−17.69, −0.32)	0.042	−10.66 (−21.46, 0.15)	0.053	−8.04 (−16.69, 0.60)	0.068	−7.92 (−16.68, 0.83)	0.075
miR-199b-5p	10.68 (−1.81, 23.17)	0.093	12.48 (1.17, 23.80)	0.031	7.31 (−5.93, 20.54)	0.276	11.45 (−0.24, 23.15)	0.055	14.98 (0.42, 29.54)	0.044	9.31 (−2.34, 20.95)	0.116	9.96 (−1.83, 21.74)	0.097
miR-136-5p	18.89 (0.51, 37.28)	0.044	20.04 (3.39, 36.70)	0.019	19.54 (0.06, 39.03)	0.049	19.37 (2.15, 36.58)	0.028	24.31 (2.88, 45.73)	0.027	18.40 (1.26, 35.54)	0.036	19.55 (2.20, 36.90)	0.028
let-7e-5p	5.29 (−2.21, 12.80)	0.165	6.87 (0.07, 13.67)	0.048	3.06 (−4.89, 11.01)	0.447	7.17 (0.14, 14.20)	0.046	9.36 (0.61, 18.11)	0.036	7.10 (0.10, 14.10)	0.047	6.57 (−0.51, 13.65)	0.069
let-7b-5p	−7.17 (−15.64, 1.29)	0.096	−8.35 (−16.02, −0.69)	0.033	−2.94 (−11.91, 6.02)	0.517	−8.72 (−16.64, −0.80)	0.031	−8.16 (−18.02, 1.70)	0.104	−6.52 (−14.40, 1.37)	0.104	−6.86 (−14.84, 1.13)	0.092
**7-year-olds**														
miR-495-3p	20.71 (0.67, 40.75)	0.043	25.52 (3.32, 47.73)	0.025	23.61 (3.33, 43.89)	0.023	16.21 (−2.84, 35.25)	0.095	20.36 (−0.35, 41.07)	0.054	23.59 (3.10, 44.09)	0.024		

This table shows only the miRNAs with suggestive associations with blood cell composition, where *p*-values were less than 0.05. Here, *p*-values < 0.05 are bolded. None of the associations remained significant after controlling for the false discovery rate (FDR) (FDR-adjusted *p*-values were > 0.05). NK: natural killer cells. nRBC: nucleated red blood cell. CI: confidence interval.

**Table 4:** Differential expression analysis of miRNAs in newborns compared to 7-year-old children.

miRNA Name	logFC *	FC	log_2_ Mean Expression	FDR-Adjusted *p*-Value
miR-1260b	−2.77	−6.84	7.66	**7.29 × 10^−34^**
let-7e-5p	−2.67	−6.36	8.08	**2.47 × 10^−83^**
miR-6511a-3p	−2.10	−4.29	6.34	**6.14 × 10^−35^**
miR-19a-3p	−2.06	−4.16	13.43	**2.95 × 10^−41^**
miR-1254	−1.95	−3.86	4.80	**2.94 × 10^−20^**
miR-425-5p	−1.81	−3.50	12.61	**1.24 × 10^−40^**
miR-200a-3p	−1.76	−3.39	6.88	**3.86 × 10^−52^**
miR-454-3p	−1.67	−3.18	11.57	**1.16 × 10^−31^**
miR-766-3p	−1.65	−3.14	7.14	**1.28 × 10^−23^**
miR-142-5p	−1.56	−2.95	7.82	**3.74 × 10^−32^**
miR-548e-5p	−1.47	−2.76	6.17	**4.32 × 10^−29^**
miR-103a-3p	−1.31	−2.49	5.67	**4.52 × 10^−17^**
miR-141-3p	−1.15	−2.22	7.32	**1.53 × 10^−18^**
miR-146a-5p	−1.10	−2.14	11.07	**2.99 × 10^−24^**
miR-335-5p	−1.09	−2.13	7.94	**6.37 × 10^−18^**
miR-26a-5p	−1.02	−2.03	13.25	**6.30 × 10^−16^**
let-7c-5p	−0.87	−1.83	11.43	**4.65 × 10^−12^**
miR-664a-3p	−0.86	−1.81	9.59	**1.62 × 10^−15^**
miR-130a-3p	−0.82	−1.77	13.12	**2.02 × 10^−18^**
miR-107	−0.82	−1.77	12.45	**5.57 × 10^−16^**
miR-130b-3p	−0.82	−1.76	8.53	**3.18 × 10^−17^**
miR-1275	−0.69	−1.61	6.54	**1.82 × 10^−8^**
miR-25-3p	−0.57	−1.49	16.60	**1.06 × 10^−4^**
miR-26b-5p	−0.57	−1.48	15.40	**3.12 × 10^−6^**
miR-4461	−0.48	−1.39	7.20	**1.53 × 10^−6^**
miR-301a-3p	−0.33	−1.25	10.03	**2.77 × 10^−3^**
let-7d-5p	−0.25	−1.19	14.81	**1.83 × 10^−2^**
miR-1289	−0.24	−1.18	8.01	**2.30 × 10^−2^**
miR-92a-3p	−0.16	−1.11	13.24	3.95 × 10^−1^
let-7b-5p	−0.10	−1.07	16.38	4.18 × 10^−1^
miR-185-5p	−0.10	−1.07	13.29	4.18 × 10^−1^
miR-223-3p	0.04	1.03	18.40	6.96 × 10^−1^
miR-199a-5p	0.14	1.10	10.85	3.47 × 10^−1^
miR-505-3p	0.20	1.15	7.43	5.84 × 10^−2^
miR-139-5p	0.34	1.26	5.44	6.93 × 10^−2^
miR-199b-5p	0.45	1.36	8.72	**4.00 × 10^−4^**
miR-92b-3p	0.55	1.46	4.59	**3.10 × 10^−3^**
miR-150-5p	0.58	1.49	17.72	**3.26 × 10^−6^**
miR-377-3p	0.63	1.55	6.94	**2.77 × 10^−3^**
miR-136-5p	0.83	1.78	7.92	**8.28 × 10^−5^**
miR-495-3p	1.05	2.07	8.22	**2.37 × 10^−9^**
miR-371b-5p	1.17	2.25	4.67	**3.27 × 10^−13^**
miR-616-3p	1.19	2.28	5.71	**2.37 × 10^−9^**

* Here, logFC is the log fold-change in miRNA expression for newborns compared to 7-year-old children. This model adjusted for cell composition (cd8+T, cd4+T, NK cells, B cells, monocytes, and granulocytes). FDR-adjusted *p*-values < 0.05 are bolded. FC: fold-change.

## Supplementary Materials

The following are available online at http://www.mdpi.com/1660-4601/16/4/524/s1, Table S1: Blood cell proportions estimated by *minfi* in cord and 7-year-old blood. Table S2: Regression model of blood cell composition on cord blood miRNA expression. Table S3: Regression model of blood cell composition on 7-year-old blood miRNA expression. Table S4: Differential expression analysis by sex in newborns and 7-year-olds. Table S5: Partial correlation of miRNAs between newborns and 7-year-olds, controlling for cell composition. Table S6: Significantly enriched GO terms related to miRNAs differentially expressed between newborns and 7-year-old children. Figure S1: PCA analysis. Cumulative variance explained by each principal component.
