# Supplementary material for: Age-Related Differences in miRNA Expression in Mexican-American Newborns and Children
Source: Int J Environ Res Public Health. 2019 Feb 13;16(4):524. doi: 10.3390/ijerph16040524 (PMC6406617; doi:10.3390/ijerph16040524)

Supplemental Tables and Figures

Supplemental Table S1. Blood cell proportions estimated by *minfi* in cord* and 7-year old blood.

	Newborns				7-year olds			
	N	Mean (%)	min	max	N	Mean (%)	min	max
CD8+ T	120	9.2	0.0	21.8	131	11.4	0.8	24.1
CD4+ T	120	10.2	0.0	36.1	131	16.8	4.8	31.8
NK cells	120	2.3	0.0	9.6	131	4.2	0.0	18.7
B cells	120	8.0	0.0	20.3	131	11.0	2.7	21.8
Monocytes	120	9.3	0.0	18.5	131	6.1	1.1	16.0
Granulocytes	120	47.4	4.2	69.7	131	50.1	25.4	79.7
nRBCs	120	16.6	3.3	93.6	0	0	0	0

*minfi estimates were based on a cord blood reference population (Bakulski et al.).

Supplemental Table S2. Regression model of blood cell composition on cord blood miRNA expression.

	cd8+T		cd4+T		NK Cells		B Cells		Monocytes		Granulocytes		nRBCs	
miRNA name	β(95%CI)	p-value	β(95%CI)	p-value	β(95%CI)	p-value	β(95%CI)	p-value	β(95%CI)	p-value	β(95%CI)	p-value	β(95%CI)	p-value
miR-92b-3p	7.28(-9.77,24.32)	0.399	7.63(-7.81,23.07)	0.330	11.3(-6.75,29.36)	0.217	10.22(-5.74,26.17)	0.207	8.62(-11.24,28.48)	0.391	9.53(-6.35,25.41)	0.237	7.88(-8.2,23.96)	0.333
miR-92a-3p	-5.06(-17.04,6.91)	0.404	-9.16(-20,1.69)	0.097	-6.17(-18.85,6.52)	0.337	-7.82(-19.03,3.39)	0.170	-9.88(-23.83,4.07)	0.163	-6.16(-17.32,4.99)	0.276	-7.74(-19.04,3.55)	0.177
miR-766-3p	6.68(-7.29,20.65)	0.345	5.11(-7.55,17.76)	0.426	0.42(-14.38,15.22)	0.955	5.64(-7.44,18.72)	0.395	3.07(-13.21,19.35)	0.709	5.27(-7.75,18.29)	0.424	4.18(-9,17.36)	0.531
miR-664a-3p	1.56(-7.74,10.86)	0.740	0.59(-7.84,9.02)	0.890	0.48(-9.38,10.33)	0.924	1.89(-6.82,10.59)	0.669	-0.14(-10.98,10.7)	0.980	1.52(-7.15,10.19)	0.729	0.89(-7.89,9.67)	0.841
miR-6511a-3p	6.9(-9.39,23.19)	0.403	7.53(-7.23,22.28)	0.314	3.36(-13.9,20.62)	0.700	7.01(-8.24,22.26)	0.364	8.62(-10.36,27.61)	0.370	7.55(-7.63,22.74)	0.326	6.88(-8.5,22.25)	0.377
miR-616-3p	3.35(-16.15,22.85)	0.734	7.02(-10.64,24.68)	0.432	6.51(-14.15,27.16)	0.534	8.18(-10.07,26.44)	0.376	8.63(-14.09,31.35)	0.453	4.27(-13.9,22.44)	0.643	4.82(-13.58,23.21)	0.605
miR-548e-5p	5.14(-8.19,18.48)	0.446	5.6(-6.48,17.68)	0.360	7.41(-6.71,21.54)	0.301	7.38(-5.1,19.86)	0.244	1.16(-14.38,16.69)	0.883	5.27(-7.15,17.7)	0.402	5.16(-7.42,17.74)	0.418
miR-505-3p	6.6(-6.23,19.44)	0.310	10.38(-1.24,22.01)	0.080	5.81(-7.79,19.41)	0.399	11.85(-0.17,23.86)	0.053	14.32(-0.64,29.28)	0.060	8.56(-3.41,20.52)	0.159	9.43(-2.68,21.54)	0.126
miR-495-3p	12.38(-4.25,29.01)	0.143	16.39(1.33,31.46)	**0.033**	14.13(-3.48,31.75)	0.115	13.1(-2.47,28.67)	0.098	19.66(0.28,39.04)	**0.047**	13.21(-2.29,28.71)	0.094	14.62(-1.07,30.31)	0.068
miR-454-3p	4.35(-4.04,12.75)	0.307	1.34(-6.27,8.94)	0.729	2.82(-6.07,11.72)	0.531	1.06(-6.8,8.92)	0.789	3.78(-6,13.57)	0.445	3.26(-4.57,11.08)	0.411	3.75(-4.17,11.67)	0.351
miR-4461	2.25(-10.24,14.74)	0.722	3.27(-8.04,14.58)	0.568	5.77(-7.46,19)	0.389	3.98(-7.71,15.67)	0.501	-1.5(-16.05,13.05)	0.838	4.5(-7.14,16.14)	0.445	3.11(-8.67,14.89)	0.602
miR-425-5p	-3.9(-11.59,3.78)	0.316	-6.07(-13.03,0.89)	0.087	-2.64(-10.78,5.51)	0.523	-4.57(-11.77,2.62)	0.210	-5.1(-14.06,3.85)	0.261	-4.37(-11.53,2.8)	0.230	-4.06(-11.31,3.19)	0.270
miR-377-3p	16.37(-0.78,33.52)	0.061	20.27(4.74,35.8)	**0.011**	12.37(-5.8,30.53)	0.180	19.31(3.25,35.36)	**0.019**	22.68(2.69,42.66)	**0.027**	17.09(1.1,33.07)	**0.036**	19.19(3.01,35.37)	**0.021**
miR-371b-5p	4.85(-12.47,22.18)	0.580	7.36(-8.34,23.05)	0.355	6.47(-11.89,24.82)	0.486	12.13(-4.09,28.35)	0.141	8.99(-11.2,29.18)	0.380	6.78(-9.37,22.93)	0.407	6.56(-9.78,22.91)	0.428
miR-335-5p	0.36(-9.39,10.11)	0.942	2.29(-6.53,11.12)	0.608	0.83(-9.5,11.15)	0.874	1.52(-7.61,10.64)	0.742	-1.39(-12.75,9.97)	0.809	-0.4(-9.48,8.68)	0.931	-0.74(-9.94,8.46)	0.874
miR-301a-3p	6.22(-1.73,14.17)	0.124	9.01(1.81,16.21)	**0.015**	8.77(0.35,17.19)	**0.041**	6.21(-1.23,13.65)	0.101	8.8(-0.46,18.07)	0.062	8.27(0.86,15.68)	**0.029**	9.07(1.57,16.57)	**0.018**
miR-26b-5p	-6.64(-15.92,2.63)	0.159	-8.92(-17.32,-0.51)	**0.038**	-5.3(-15.12,4.53)	0.288	-9.01(-17.69,-0.32)	**0.042**	-10.66(-21.46,0.15)	0.053	-8.04(-16.69,0.6)	0.068	-7.92(-16.68,0.83)	0.075
miR-26a-5p	4.77(-3.87,13.41)	0.276	2.77(-5.05,10.6)	0.484	3.24(-5.91,12.38)	0.485	0.73(-7.36,8.81)	0.859	4.4(-5.66,14.47)	0.388	3.23(-4.82,11.28)	0.428	2.68(-5.47,10.83)	0.516
miR-25-3p	-3.36(-16.88,10.16)	0.623	-2.04(-14.29,10.21)	0.742	4.45(-9.87,18.78)	0.539	0.63(-12.03,13.29)	0.922	-1.29(-17.04,14.47)	0.872	-1.3(-13.9,11.3)	0.838	-0.25(-13.01,12.51)	0.969
miR-223-3p	-6.53(-20.61,7.56)	0.361	-6(-18.76,6.76)	0.354	-14.47(-29.39,0.45)	0.057	-5.8(-18.98,7.39)	0.386	-2.6(-19.01,13.82)	0.754	-9.23(-22.36,3.9)	0.166	-7.72(-21.01,5.57)	0.252
miR-200a-3p	1.82(-7.17,10.81)	0.689	3.37(-4.77,11.52)	0.413	2.51(-7.01,12.03)	0.603	3.22(-5.19,11.64)	0.449	4.36(-6.11,14.84)	0.411	2.27(-6.11,10.65)	0.592	2.82(-5.66,11.3)	0.511
miR-19a-3p	0.7(-8.26,9.67)	0.877	1.67(-6.45,9.78)	0.685	3.12(-6.37,12.61)	0.516	0.03(-8.36,8.42)	0.995	-0.35(-10.8,10.09)	0.947	1.08(-7.27,9.43)	0.798	2.1(-6.35,10.56)	0.623
miR-199b-5p	10.68(-1.81,23.17)	0.093	12.48(1.17,23.8)	**0.031**	7.31(-5.93,20.54)	0.276	11.45(-0.24,23.15)	0.055	14.98(0.42,29.54)	**0.044**	9.31(-2.34,20.95)	0.116	9.96(-1.83,21.74)	0.097
miR-199a-5p	3.91(-7.57,15.4)	0.501	7.06(-3.34,17.47)	0.181	0.21(-11.96,12.38)	0.973	7.76(-2.99,18.51)	0.156	7.3(-6.08,20.69)	0.282	3.73(-6.98,14.43)	0.492	4.46(-6.37,15.3)	0.416
miR-185-5p	1.49(-7.47,10.46)	0.742	1.77(-6.35,9.89)	0.666	7.96(-1.53,17.46)	0.099	0.42(-7.97,8.81)	0.921	1.85(-8.6,12.3)	0.726	2.25(-6.1,10.61)	0.594	2.61(-5.85,11.07)	0.542
miR-150-5p	-1.55(-14.43,11.32)	0.811	-5.44(-17.1,6.23)	0.358	-4.21(-17.85,9.43)	0.542	-4.07(-16.12,7.98)	0.505	-5.12(-20.12,9.88)	0.500	-4.29(-16.29,7.71)	0.481	-4.63(-16.78,7.52)	0.452
miR-146a-5p	2.91(-7.19,13)	0.569	6.06(-3.09,15.2)	0.192	3.59(-7.1,14.28)	0.508	5.49(-3.96,14.94)	0.252	4.45(-7.31,16.21)	0.455	2.87(-6.53,12.28)	0.546	3.06(-6.46,12.58)	0.525
miR-142-5p	7.6(-3.75,18.95)	0.187	6.61(-3.67,16.89)	0.206	6.38(-5.64,18.4)	0.295	4.1(-6.53,14.72)	0.447	4.63(-8.59,17.86)	0.489	6.62(-3.96,17.2)	0.218	5.75(-4.96,16.46)	0.289

miR-141-3p	6.47(-9.56,22.49)	0.426	7.61(-6.9,22.13)	0.301	2.9(-14.07,19.88)	0.735	7.8(-7.21,22.8)	0.305	5.45(-13.22,24.12)	0.564	6.72(-8.22,21.66)	0.375	6.8(-8.32,21.92)	0.375
miR-139-5p	8.22(-10.16,26.6)	0.377	11.12(-5.53,27.77)	0.188	16.13(-3.34,35.6)	0.103	16.08(-1.12,33.29)	0.067	4.39(-17.02,25.81)	0.685	13.1(-4.03,30.23)	0.132	11.69(-5.65,29.03)	0.184
miR-136-5p	18.89(0.51,37.28)	**0.044**	20.04(3.39,36.7)	**0.019**	19.54(0.06,39.03)	**0.049**	19.37(2.15,36.58)	**0.028**	24.31(2.88,45.73)	**0.027**	18.4(1.26,35.54)	**0.036**	19.55(2.2,36.9)	**0.028**
miR-130b-3p	0.24(-9.2,9.67)	0.961	2.28(-6.26,10.83)	0.597	3.07(-6.93,13.06)	0.544	2.81(-6.02,11.65)	0.529	-0.67(-11.67,10.32)	0.904	2.13(-6.66,10.93)	0.632	2.14(-6.76,11.04)	0.635
miR-130a-3p	0.12(-6.79,7.04)	0.972	1.32(-4.95,7.58)	0.678	2.28(-5.05,9.6)	0.539	2.45(-4.02,8.92)	0.455	-1.06(-9.12,7)	0.795	0.6(-5.84,7.05)	0.853	1.1(-5.42,7.63)	0.738
miR-1289	3.38(-8.92,15.69)	0.587	2.15(-8.99,13.3)	0.703	2.69(-10.34,15.72)	0.683	4.22(-7.3,15.74)	0.469	0.86(-13.48,15.2)	0.906	3.1(-8.37,14.57)	0.593	1.97(-9.64,13.58)	0.737
miR-1275	2.63(-10.86,16.13)	0.699	1.48(-10.74,13.7)	0.811	1.72(-12.57,16.01)	0.812	5.98(-6.65,18.61)	0.350	1.64(-14.08,17.36)	0.836	2.99(-9.59,15.56)	0.639	2.53(-10.2,15.26)	0.695
miR-1260b	-3.27(-20.36,13.81)	0.705	-2.64(-18.11,12.83)	0.736	-13.91(-32,4.19)	0.131	-0.4(-16.39,15.59)	0.961	-6.17(-26.08,13.73)	0.540	-3.03(-18.95,12.89)	0.707	-5.01(-21.13,11.1)	0.539
miR-1254	1.9(-18.18,21.97)	0.852	2.87(-15.31,21.05)	0.755	0.64(-20.63,21.9)	0.953	6.57(-12.23,25.36)	0.490	5.21(-18.19,28.6)	0.660	3.88(-14.83,22.59)	0.682	2.81(-16.13,21.75)	0.769
miR-107	1.42(-3.85,6.7)	0.594	0.68(-4.1,5.46)	0.779	1.26(-4.33,6.85)	0.655	-0.66(-5.6,4.28)	0.793	2.58(-3.57,8.73)	0.407	1.66(-3.26,6.58)	0.505	1.28(-3.7,6.25)	0.613
miR-103a-3p	7.16(-4.72,19.04)	0.235	7.86(-2.9,18.62)	0.151	9.14(-3.44,21.72)	0.153	10.74(-0.38,21.86)	0.058	10.15(-3.69,23.99)	0.149	8.39(-2.68,19.46)	0.136	8.62(-2.59,19.82)	0.130
let-7e-5p	5.29(-2.21,12.8)	0.165	6.87(0.07,13.67)	**0.048**	3.06(-4.89,11.01)	0.447	7.17(0.14,14.2)	**0.046**	9.36(0.61,18.11)	**0.036**	7.1(0.1,14.1)	**0.047**	6.57(-0.51,13.65)	0.069
let-7d-5p	-4.89(-14.35,4.58)	0.309	-5.79(-14.37,2.79)	0.184	-5.28(-15.31,4.75)	0.299	-5.8(-14.67,3.06)	0.197	-4.21(-15.24,6.82)	0.451	-3.99(-12.81,4.84)	0.373	-4.15(-13.08,4.78)	0.359
let-7c-5p	-1.66(-9.48,6.17)	0.676	-1.8(-8.89,5.29)	0.617	2.47(-5.82,10.76)	0.556	-2.77(-10.1,4.55)	0.455	-0.7(-9.82,8.42)	0.879	0.31(-6.98,7.61)	0.933	-0.59(-7.98,6.8)	0.875
let-7b-5p	-7.17(-15.64,1.29)	0.096	-8.35(-16.02,-0.69)	**0.033**	-2.94(-11.91,6.02)	0.517	-8.72(-16.64,-0.8)	**0.031**	-8.16(-18.02,1.7)	0.104	-6.52(-14.4,1.37)	0.104	-6.86(-14.84,1.13)	0.092

*Unadjusted p-values <0.05 are bolded. None of the associations remained significant after controlling for the FDR (FDR adjusted p-values were > 0.05.

Supplemental Table S3. Regression model of blood cell composition on 7-year old blood miRNA expression.

	cd8+T		cd4+T		NK Cells		B Cells		Monocytes		Granulocytes	
	cd8+T	cd4+T	NK Cells	B Cells	Monocytes	Granulocytes	nRBCs	p-value	β(95%CI)	p-value	β(95%CI)	p-value
miR-92a-3p	-1.39(-15.72,12.95)	0.849	-3.15(-19.03,12.73)	0.695	-3.05(-17.55,11.46)	0.678	-5.83(-19.45,7.79)	0.398	3.23(-11.58,18.05)	0.667	-4.89(-19.54,9.77)	0.511
miR-766-3p	5.76(-11.3,22.82)	0.505	5.82(-13.07,24.72)	0.543	6.66(-10.6,23.92)	0.446	6.62(-9.59,22.82)	0.420	3.13(-14.5,20.75)	0.726	6.23(-11.21,23.67)	0.481
miR-664a-3p	2.88(-5.72,11.47)	0.509	0.95(-8.57,10.47)	0.844	1.03(-7.67,9.72)	0.815	0.14(-8.03,8.3)	0.974	-0.59(-9.47,8.29)	0.896	1.47(-7.32,10.26)	0.741
miR-6511a-3p	5.48(-13.06,24.03)	0.559	7.8(-12.75,28.35)	0.454	7.86(-10.91,26.62)	0.409	8.69(-8.93,26.31)	0.331	7.71(-11.46,26.88)	0.428	5.69(-13.28,24.65)	0.554
miR-616-3p	-0.87(-23.81,22.08)	0.941	7.7(-17.72,33.13)	0.550	2.79(-20.42,26.01)	0.812	3.6(-18.2,25.4)	0.744	-2.18(-25.9,21.53)	0.856	3.32(-20.14,26.79)	0.780
miR-548e-5p	5.96(-9.6,21.53)	0.450	5.77(-11.48,23.01)	0.509	6.41(-9.34,22.15)	0.422	5.16(-9.62,19.95)	0.491	2.29(-13.8,18.37)	0.779	4.5(-11.42,20.42)	0.577
miR-505-3p	1.52(-11.79,14.83)	0.822	6.41(-8.34,21.15)	0.392	4.2(-9.26,17.67)	0.538	3.52(-9.12,16.17)	0.582	3.11(-10.65,16.86)	0.656	5.98(-7.63,19.59)	0.386
miR-495-3p	20.71(0.67,40.75)	**0.043**	25.52(3.32,47.73)	**0.025**	23.61(3.33,43.89)	**0.023**	16.21(-2.84,35.25)	0.095	20.36(-0.35,41.07)	0.054	23.59(3.1,44.09)	**0.024**
miR-454-3p	-2.97(-11.73,5.78)	0.503	-3.59(-13.29,6.11)	0.465	-2.33(-11.19,6.52)	0.603	-2.42(-10.73,5.9)	0.566	0.63(-8.41,9.68)	0.890	-3.97(-12.92,4.98)	0.382
miR-4461	6.36(-8.37,21.09)	0.394	6.22(-10.11,22.54)	0.452	7.56(-7.34,22.47)	0.317	5.39(-8.61,19.39)	0.447	6.78(-8.44,22.01)	0.380	5.44(-9.63,20.5)	0.476
miR-425-5p	-6(-15.04,3.05)	0.192	-8.2(-18.22,1.82)	0.108	-6.9(-16.05,2.26)	0.138	-6.25(-14.84,2.35)	0.153	-6.89(-16.24,2.46)	0.147	-7.77(-17.02,1.48)	0.099
miR-377-3p	5.53(-17.1,28.17)	0.629	9.61(-15.47,34.7)	0.450	9.27(-13.64,32.18)	0.425	6.98(-14.52,28.49)	0.522	1.55(-21.85,24.95)	0.896	9.62(-13.54,32.77)	0.413
miR-371b-5p	7.77(-12.04,27.58)	0.439	10.21(-11.74,32.16)	0.359	7.43(-12.62,27.47)	0.465	7.71(-11.11,26.53)	0.419	9.26(-11.21,29.74)	0.372	9.43(-10.83,29.69)	0.359
miR-335-5p	1.13(-11.09,13.35)	0.856	0.67(-12.87,14.21)	0.922	0.15(-12.22,12.51)	0.981	0.27(-11.34,11.88)	0.963	-0.44(-13.07,12.19)	0.945	1.56(-10.93,14.06)	0.805
miR-301a-3p	4.17(-4.5,12.85)	0.343	8.1(-1.51,17.71)	0.098	5.09(-3.69,13.86)	0.254	5.86(-2.38,14.1)	0.162	5.41(-3.56,14.37)	0.235	6.42(-2.45,15.29)	0.154
miR-26b-5p	-3.03(-14.27,8.22)	0.595	-4.93(-17.39,7.53)	0.435	-3.69(-15.07,7.69)	0.522	-2.55(-13.24,8.13)	0.637	-3.09(-14.71,8.53)	0.599	-4.59(-16.09,6.91)	0.431
miR-26a-5p	4.16(-6.01,14.34)	0.419	3.41(-7.86,14.68)	0.551	2.92(-7.37,13.22)	0.575	2.94(-6.73,12.6)	0.549	3.04(-7.48,13.55)	0.568	3.99(-6.41,14.4)	0.449
miR-25-3p	-6.35(-22.19,9.49)	0.429	-4.29(-21.85,13.26)	0.629	-4.89(-20.92,11.14)	0.547	-3.9(-18.95,11.15)	0.609	-3.89(-20.26,12.49)	0.639	-5.57(-21.77,10.63)	0.497
miR-223-3p	-5.04(-21.42,11.35)	0.544	-8.47(-26.62,9.68)	0.357	-8.4(-24.97,8.18)	0.318	-8.18(-23.74,7.39)	0.301	-11.01(-27.95,5.92)	0.200	-3.51(-20.27,13.24)	0.679
miR-200a-3p	0.74(-10.49,11.97)	0.897	0.33(-12.12,12.77)	0.959	0.38(-10.99,11.74)	0.948	0.69(-9.98,11.36)	0.899	-2.32(-13.92,9.29)	0.693	2.28(-9.2,13.77)	0.695
miR-19a-3p	-0.97(-11.63,9.69)	0.857	-0.15(-11.96,11.66)	0.980	0.33(-10.46,11.11)	0.952	3.04(-7.08,13.17)	0.553	0.18(-10.83,11.2)	0.974	-0.65(-11.54,10.25)	0.907
miR-199b-5p	2.06(-11.37,15.49)	0.762	3.15(-11.73,18.03)	0.676	3.49(-10.1,17.07)	0.613	5.37(-7.39,18.13)	0.407	-0.99(-14.87,12.89)	0.888	6.56(-7.17,20.29)	0.346
miR-199a-5p	2.07(-11.65,15.79)	0.766	4.47(-10.73,19.67)	0.562	4.88(-9,18.76)	0.488	2.96(-10.08,15.99)	0.654	2.51(-11.67,16.69)	0.727	4.54(-9.49,18.57)	0.523
miR-185-5p	0.16(-8.71,9.03)	0.972	2.89(-6.93,12.72)	0.561	2.92(-6.05,11.89)	0.521	4.09(-4.34,12.51)	0.339	2.88(-6.28,12.04)	0.535	1.1(-7.96,10.17)	0.810
miR-150-5p	-2.89(-16.46,10.67)	0.674	-8.18(-23.21,6.85)	0.283	-8.79(-22.52,4.93)	0.207	-8.45(-21.33,4.44)	0.197	-12.46(-26.48,1.56)	0.081	-8.73(-22.6,5.14)	0.215
miR-146a-5p	3.24(-7.23,13.71)	0.541	4.28(-7.32,15.88)	0.467	4.29(-6.31,14.88)	0.425	3.48(-6.47,13.43)	0.490	2.42(-8.4,13.24)	0.659	4.09(-6.62,14.79)	0.451
miR-142-5p	10.85(-2.76,24.46)	0.117	12.53(-2.55,27.62)	0.102	10.48(-3.29,24.25)	0.135	12.12(-0.81,25.06)	0.066	9.11(-4.96,23.18)	0.202	11.26(-2.66,25.18)	0.112
miR-141-3p	7.11(-10.32,24.53)	0.421	10.93(-8.38,30.23)	0.265	9.71(-7.92,27.34)	0.278	11.51(-5.04,28.06)	0.171	4.96(-13.05,22.96)	0.587	9.66(-8.16,27.48)	0.285
miR-139-5p	9.98(-12.72,32.69)	0.386	16.17(-8.99,41.33)	0.206	13.05(-9.93,36.03)	0.263	12.4(-9.17,33.97)	0.257	13.02(-10.45,36.49)	0.274	12.91(-10.31,36.13)	0.273
miR-136-5p	18.91(-4.26,42.08)	0.109	21.64(-4.04,47.31)	0.098	22.2(-1.24,45.65)	0.063	20.25(-1.76,42.27)	0.071	16.78(-7.16,40.73)	0.168	21.55(-2.14,45.25)	0.074
miR-130b-3p	2.75(-7.98,13.48)	0.613	1.92(-9.97,13.81)	0.750	2.72(-8.14,13.58)	0.621	2.92(-7.27,13.12)	0.571	1.24(-9.85,12.33)	0.825	2.4(-8.57,13.38)	0.665
miR-130a-3p	-2.08(-9.14,4.97)	0.560	-1.57(-9.39,6.25)	0.692	-1.8(-8.94,5.34)	0.618	-1.51(-8.22,5.19)	0.656	-3.11(-10.4,4.19)	0.401	-1.75(-8.97,5.46)	0.631

miR-1289	-4.74(-19.06,9.57)	0.513	-8.9(-24.76,6.97)	0.269	-8.48(-22.97,6)	0.249	-6.38(-19.99,7.22)	0.355	-9.58(-24.37,5.22)	0.203	-8.09(-22.73,6.55)	0.276
miR-1275	1.02(-14.21,16.25)	0.894	1.61(-15.27,18.48)	0.851	1.86(-13.55,17.27)	0.811	0.45(-14.02,14.92)	0.951	-2.14(-17.88,13.6)	0.788	0.46(-15.11,16.04)	0.953
miR-1260b	4.28(-13.77,22.32)	0.640	5.73(-14.26,25.72)	0.571	5.54(-12.71,23.8)	0.549	5.2(-11.95,22.34)	0.550	6.07(-12.58,24.72)	0.520	5.01(-13.44,23.46)	0.592
miR-1254	3.58(-20.7,27.85)	0.771	0.66(-26.24,27.56)	0.961	0.13(-24.44,24.69)	0.992	3.23(-19.84,26.29)	0.782	-2.06(-27.16,23.03)	0.871	3.17(-21.65,28)	0.801
miR-107	-0.25(-6.44,5.95)	0.937	-0.33(-7.2,6.53)	0.924	-0.18(-6.45,6.09)	0.955	-0.08(-5.97,5.8)	0.978	-0.08(-6.48,6.32)	0.980	-0.29(-6.63,6.04)	0.928
miR-103a-3p	-2.66(-18.89,13.58)	0.747	0.31(-17.68,18.29)	0.973	-1.02(-17.44,15.41)	0.903	3.49(-11.93,18.92)	0.655	2.35(-14.43,19.13)	0.782	-0.59(-17.2,16.01)	0.944
let-7e-5p	2.83(-7.09,12.76)	0.573	5.85(-5.15,16.84)	0.295	3.89(-6.15,13.94)	0.444	3.56(-5.87,12.99)	0.456	6.1(-4.15,16.36)	0.241	5.66(-4.49,15.81)	0.272
let-7d-5p	0.43(-7.86,8.72)	0.918	-0.87(-10.06,8.32)	0.851	-0.28(-8.67,8.11)	0.947	-0.73(-8.61,7.15)	0.855	1.16(-7.41,9.74)	0.789	-0.76(-9.24,7.73)	0.860
let-7c-5p	2.82(-7.89,13.54)	0.603	4.07(-7.8,15.95)	0.498	3.93(-6.92,14.77)	0.475	1.72(-8.46,11.9)	0.739	6.62(-4.45,17.7)	0.239	2.53(-8.43,13.49)	0.649
let-7b-5p	-2.02(-13.16,9.12)	0.721	-1.75(-14.09,10.59)	0.779	-0.86(-12.13,10.41)	0.880	-1.64(-12.23,8.94)	0.759	1.04(-10.47,12.56)	0.858	-2.86(-14.26,8.53)	0.619

*Unadjusted p-values <0.05 are bolded. None of the associations remained significant after controlling for the FDR (FDR adjusted p-values were > 0.05.

Supplemental Table S4. Differential expression analysis by sex in newborns and 7-year olds.

	Newborns (cord blood)				7- year olds			
miRNA name	logFC	FC	Log2 Mean Expression	FDR Adjusted P-value	logFC	FC	Log2 Mean Expression	FDR Adjusted P-value
hsa-miR-1260b	-0.42	-1.34	6.24	0.63	0.11	1.08	8.93	0.80
hsa-miR-664a-3p	-0.26	-1.20	9.12	0.63	-0.04	-1.03	10.01	0.80
hsa-miR-548e-5p	-0.22	-1.17	5.31	0.63	0.06	1.04	6.95	0.80
hsa-miR-6511a-3p	-0.22	-1.17	5.22	0.63	0.06	1.04	7.34	0.82
hsa-miR-136-5p	-0.21	-1.16	8.36	0.63	0.24	1.18	7.53	0.54
hsa-miR-26b-5p	-0.21	-1.15	15.07	0.63	-0.12	-1.09	15.70	0.54
hsa-miR-766-3p	-0.20	-1.15	6.27	0.63	0.10	1.07	7.91	0.80
hsa-miR-150-5p	-0.20	-1.15	17.91	0.63	0.22	1.16	17.54	0.24
hsa-miR-92b-3p	-0.20	-1.15	4.94	0.63	-0.15	-1.11	4.28	0.68
hsa-miR-616-3p	-0.20	-1.15	6.27	0.63	0.34	1.26	5.20	0.36
hsa-miR-26a-5p	-0.20	-1.15	12.75	0.63	-0.09	-1.06	13.70	0.76
hsa-let-7d-5p	-0.20	-1.15	14.71	0.63	-0.23	-1.17	14.90	0.17
hsa-miR-142-5p	-0.19	-1.14	6.99	0.63	0.01	1.01	8.56	0.93
hsa-miR-92a-3p	-0.19	-1.14	13.21	0.63	-0.08	-1.06	13.26	0.80
hsa-miR-454-3p	-0.18	-1.14	10.71	0.63	-0.18	-1.13	12.35	0.36
hsa-miR-141-3p	-0.18	-1.13	6.68	0.63	0.15	1.11	7.90	0.47
hsa-miR-495-3p	-0.17	-1.13	8.82	0.63	-0.27	-1.21	7.68	0.36
hsa-miR-425-5p	-0.15	-1.11	11.68	0.63	0.04	1.03	13.44	0.82
hsa-miR-19a-3p	-0.15	-1.11	12.31	0.63	0.10	1.07	14.43	0.68
hsa-miR-130b-3p	-0.14	-1.10	8.11	0.63	0.14	1.10	8.90	0.36
hsa-miR-103a-3p	-0.11	-1.08	5.00	0.75	-0.19	-1.14	6.27	0.46
hsa-miR-130a-3p	-0.11	-1.08	12.67	0.63	-0.04	-1.03	13.52	0.80
hsa-miR-1289	-0.11	-1.08	7.86	0.63	0.22	1.17	8.14	0.21
hsa-miR-377-3p	-0.11	-1.08	7.23	0.79	0.02	1.01	6.68	0.93
hsa-let-7e-5p	-0.10	-1.07	6.77	0.63	-0.19	-1.14	9.25	0.22
hsa-miR-301a-3p	-0.09	-1.07	9.84	0.75	-0.17	-1.13	10.20	0.33
hsa-miR-199a-5p	-0.08	-1.06	10.90	0.79	-0.18	-1.13	10.81	0.46
hsa-miR-223-3p	-0.08	-1.05	18.49	0.75	0.17	1.13	18.32	0.33
hsa-miR-335-5p	-0.06	-1.04	7.36	0.79	0.19	1.14	8.45	0.36
hsa-miR-107	-0.06	-1.04	12.04	0.79	-0.11	-1.08	12.82	0.54
hsa-miR-1275	-0.04	-1.03	6.15	0.83	0.09	1.06	6.89	0.76
hsa-miR-200a-3p	-0.01	-1.00	6.00	0.97	0.04	1.03	7.67	0.80
hsa-miR-1254	0.00	1.00	3.86	1.00	0.28	1.21	5.63	0.43
hsa-miR-146a-5p	0.01	1.01	10.46	0.95	-0.06	-1.04	11.61	0.80
hsa-let-7c-5p	0.04	1.03	11.03	0.83	-0.30	-1.23	11.79	0.17
hsa-let-7b-5p	0.04	1.03	16.35	0.83	-0.28	-1.21	16.41	0.18
hsa-miR-185-5p	0.06	1.04	13.24	0.79	-0.03	-1.02	13.34	0.88
hsa-miR-199b-5p	0.07	1.05	8.97	0.79	-0.02	-1.01	8.48	0.88
hsa-miR-4461	0.07	1.05	6.96	0.75	0.03	1.02	7.41	0.88
hsa-miR-139-5p	0.08	1.06	5.59	0.79	0.06	1.04	5.31	0.86
hsa-miR-371b-5p	0.09	1.06	5.33	0.79	-0.03	-1.02	4.09	0.88
hsa-miR-25-3p	0.17	1.12	16.30	0.63	-0.33	-1.26	16.87	0.17
hsa-miR-505-3p	0.17	1.13	7.62	0.63	-0.16	-1.12	7.27	0.37

FC- fold change

*logFC is for the difference in miRNA expression in girls compared to boys at birth or 7 years of age. This model adjusted for cell composition (cd8+T, cd4+T, NK cells, B cells, monocytes, and granulocytes).

Supplemental Table S5. Partial correlation of miRNAs between newborns and 7-year olds controlling for cell composition.

miRNA name	rho	p-value
miR-26b-5p	-0.23	0.08
miR-103a-3p	-0.21	0.11
miR-25-3p	-0.20	0.13
miR-150-5p	-0.19	0.14
miR-130a-3p	-0.18	0.17
miR-130b-3p	-0.18	0.18
miR-548e-5p	-0.14	0.29
miR-616-3p	-0.13	0.33
miR-6511a-3p	-0.12	0.35
miR-425-5p	-0.12	0.35
miR-1254	-0.12	0.38
miR-1289	-0.11	0.42
miR-1260b	-0.11	0.43
miR-4461	-0.10	0.44
let-7b-5p	-0.08	0.55
miR-185-5p	-0.08	0.57
miR-107	-0.08	0.57
let-7e-5p	-0.07	0.58
miR-19a-3p	-0.07	0.59
miR-454-3p	-0.07	0.61
miR-1275	-0.07	0.62
miR-92b-3p	-0.07	0.62
miR-664a-3p	-0.04	0.77
let-7d-5p	-0.01	0.92
miR-301a-3p	-0.01	0.94
miR-26a-5p	0.01	0.96
miR-371b-5p	0.01	0.96
miR-141-3p	0.01	0.94
miR-766-3p	0.01	0.93
miR-335-5p	0.01	0.91
miR-505-3p	0.02	0.88
miR-142-5p	0.03	0.81
miR-199b-5p	0.03	0.80
miR-223-3p	0.04	0.77
miR-139-5p	0.06	0.65
miR-495-3p	0.09	0.48
miR-136-5p	0.14	0.30
miR-377-3p	0.14	0.29
let-7c-5p	0.14	0.28
miR-200a-3p	0.16	0.23
miR-92a-3p	0.16	0.23
miR-199a-5p	0.17	0.19
miR-146a-5p	0.20	0.14

Supplemental Table S6. Significantly enriched GO terms related to miRNAs differentially expressed between newborns and 7-year old children.

GO Term ID	GO Term Category	p-value*	#genes	#miRNAs
GO:0043226	organelle	6.89E-311	4841	39
GO:0034641	cellular nitrogen compound metabolic process	2.19E-175	2419	39
GO:0043167	ion binding	1.16E-174	3008	39
GO:0009058	biosynthetic process	3.57E-115	2009	39
GO:0006464	cellular protein modification process	5.21E-85	1216	39
GO:0010467	gene expression	1.64E-57	329	38
GO:0044281	small molecule metabolic process	7.53E-54	1115	39
GO:0003674	molecular_function	3.53E-51	7545	39
GO:0001071	nucleic acid binding transcription factor activity	4.39E-50	554	39
GO:0048011	neurotrophin TRK receptor signaling pathway	8.38E-48	166	39
GO:0005575	cellular_component	1.50E-40	7585	39
GO:0044403	symbiosis, encompassing mutualism through parasitism	2.12E-37	278	38
GO:0009056	catabolic process	6.13E-37	906	39
GO:0019899	enzyme binding	9.09E-37	649	39
GO:0016032	viral process	1.04E-36	249	38
GO:0022607	cellular component assembly	9.55E-36	646	39
GO:0043234	protein complex	2.79E-35	1726	39
GO:0005829	cytosol	1.27E-34	1292	39
GO:0000988	protein binding transcription factor activity	8.19E-33	275	39
GO:0038095	Fc-epsilon receptor signaling pathway	1.15E-30	100	37
GO:0000278	mitotic cell cycle	1.77E-29	208	39
GO:0005654	nucleoplasm	1.42E-28	582	39
GO:0061024	membrane organization	1.39E-27	305	39
GO:0007596	blood coagulation	9.60E-26	231	38
GO:0065003	macromolecular complex assembly	5.35E-23	422	39
GO:0044267	cellular protein metabolic process	1.93E-21	213	39
GO:0034655	nucleobase-containing compound catabolic process	1.24E-20	417	39
GO:0008092	cytoskeletal protein binding	2.84E-19	375	39
GO:0007173	epidermal growth factor receptor signaling pathway	1.58E-18	121	38
GO:0006950	response to stress	1.35E-17	971	39
GO:0043687	post-translational protein modification	6.13E-17	89	38
GO:0030234	enzyme regulator activity	2.39E-16	391	39
GO:0008219	cell death	4.45E-16	421	39
GO:0044255	cellular lipid metabolic process	7.35E-16	82	38
GO:0007268	synaptic transmission	5.84E-15	208	39
GO:0007267	cell-cell signaling	1.98E-14	311	39
GO:0008150	biological_process	2.58E-14	7193	39
GO:0006461	protein complex assembly	1.60E-13	347	39
GO:0034166	toll-like receptor 10 signaling pathway	4.28E-13	39	31
GO:0038096	Fc-gamma receptor signaling pathway involved in phagocytosis	1.02E-12	43	33
GO:0038123	toll-like receptor TLR1:TLR2 signaling pathway	1.68E-12	40	31
GO:0038124	toll-like receptor TLR6:TLR2 signaling pathway	1.68E-12	40	31
GO:0035666	TRIF-dependent toll-like receptor signaling pathway	3.15E-12	42	34

GO:0030168	platelet activation	4.05E-12	102	36
GO:0008543	fibroblast growth factor receptor signaling pathway	9.73E-12	104	37
GO:0006921	cellular component disassembly involved in execution phase of apoptosis	1.55E-11	31	25
GO:0030203	glycosaminoglycan metabolic process	6.65E-11	58	35
GO:0034162	toll-like receptor 9 signaling pathway	7.67E-11	42	31
GO:0006351	transcription, DNA-templated	1.59E-10	1111	39
GO:0034146	toll-like receptor 5 signaling pathway	1.60E-10	39	31
GO:0006766	vitamin metabolic process	2.43E-10	44	31
GO:0002756	MyD88-independent toll-like receptor signaling pathway	5.04E-10	42	34
GO:0006767	water-soluble vitamin metabolic process	6.39E-10	40	27
GO:0034330	cell junction organization	1.96E-09	82	35
GO:0034329	cell junction assembly	2.08E-09	38	32
GO:0018279	protein N-linked glycosylation via asparagine	3.51E-09	55	33
GO:0000086	G2/M transition of mitotic cell cycle	4.98E-09	80	33
GO:0048870	cell motility	5.24E-09	260	39
GO:0048015	phosphatidylinositol-mediated signaling	5.52E-09	74	35
GO:0006259	DNA metabolic process	6.41E-09	333	39
GO:0006112	energy reserve metabolic process	7.80E-09	54	35
GO:0005815	microtubule organizing center	1.43E-08	217	39
GO:0034138	toll-like receptor 3 signaling pathway	2.29E-08	43	34
GO:0002224	toll-like receptor signaling pathway	2.29E-08	58	36
GO:0007411	axon guidance	3.54E-08	221	39
GO:0034142	toll-like receptor 4 signaling pathway	6.68E-08	50	35
GO:0034134	toll-like receptor 2 signaling pathway	7.05E-08	41	31
GO:0051403	stress-activated MAPK cascade	2.58E-07	32	31
GO:0002376	immune system process	2.58E-07	648	39
GO:0006367	transcription initiation from RNA polymerase II promoter	2.62E-07	107	38
GO:0002576	platelet degranulation	3.84E-07	38	32
GO:0006369	termination of RNA polymerase II transcription	6.24E-07	30	27
GO:0030204	chondroitin sulfate metabolic process	7.09E-07	28	31
GO:0016071	mRNA metabolic process	7.48E-07	86	37
GO:0006661	phosphatidylinositol biosynthetic process	2.62E-06	37	28
GO:0043647	inositol phosphate metabolic process	3.46E-06	27	31
GO:0003723	RNA binding	3.60E-06	757	39
GO:0009653	anatomical structure morphogenesis	6.08E-06	56	33
GO:0016070	RNA metabolic process	6.69E-06	98	37
GO:0031124	mRNA 3'-end processing	7.45E-06	26	26
GO:0006987	activation of signaling protein activity involved in unfolded protein response	7.99E-06	32	31
GO:0070423	nucleotide-binding oligomerization domain containing signaling pathway	8.88E-06	17	18
GO:0097193	intrinsic apoptotic signaling pathway	9.52E-06	39	34
GO:0006892	post-Golgi vesicle-mediated transport	1.05E-05	29	32
GO:0006325	chromatin organization	1.67E-05	62	36
GO:0006790	sulfur compound metabolic process	1.89E-05	122	36
GO:0060397	JAK-STAT cascade involved in growth hormone signaling pathway	2.04E-05	17	20

GO:0007202	activation of phospholipase C activity	3.31E-05	35	35
GO:0002755	MyD88-dependent toll-like receptor signaling pathway	4.01E-05	45	33
GO:0055086	nucleobase-containing small molecule metabolic process	6.62E-05	31	29
GO:0050900	leukocyte migration	6.89E-05	54	35
GO:0006644	phospholipid metabolic process	6.89E-05	76	36
GO:0008286	insulin receptor signaling pathway	7.26E-05	80	35
GO:0035872	nucleotide-binding domain, leucine rich repeat containing receptor signaling pathway	8.20E-05	23	25
GO:0061418	regulation of transcription from RNA polymerase II promoter in response to hypoxia	1.12E-04	18	26
GO:0030705	cytoskeleton-dependent intracellular transport	1.17E-04	54	34
GO:0030674	protein binding, bridging	1.51E-04	75	37
GO:1900740	positive regulation of protein insertion into mitochondrial membrane involved in apoptotic signaling pathway	1.55E-04	19	29
GO:0030198	extracellular matrix organization	2.35E-04	158	36
GO:0008645	hexose transport	3.32E-04	21	26
GO:0022857	transmembrane transporter activity	3.86E-04	422	39
GO:0010827	regulation of glucose transport	4.73E-04	17	23
GO:0007077	mitotic nuclear envelope disassembly	6.08E-04	19	23
GO:0032182	small conjugating protein binding	7.78E-04	45	33
GO:0050690	regulation of defense response to virus by virus	1.18E-03	16	22
GO:0051056	regulation of small GTPase mediated signal transduction	1.33E-03	95	34
GO:0006928	cellular component movement	1.40E-03	50	36
GO:0022617	extracellular matrix disassembly	1.66E-03	44	26
GO:0000288	nuclear-transcribed mRNA catabolic process, deadenylation-dependent decay	1.92E-03	29	32
GO:0022618	ribonucleoprotein complex assembly	3.02E-03	65	34
GO:0097190	apoptotic signaling pathway	3.26E-03	59	35
GO:0007010	cytoskeleton organization	4.04E-03	272	39
GO:0015949	nucleobase-containing small molecule interconversion	4.34E-03	10	17
GO:0035338	long-chain fatty-acyl-CoA biosynthetic process	4.34E-03	10	22
GO:0045216	cell-cell junction organization	4.34E-03	38	33
GO:0051439	regulation of ubiquitin-protein ligase activity involved in mitotic cell cycle	5.49E-03	29	30
GO:0007179	transforming growth factor beta receptor signaling pathway	6.38E-03	86	37
GO:0016192	vesicle-mediated transport	6.38E-03	413	39
GO:0006091	generation of precursor metabolites and energy	7.12E-03	126	37
GO:0008289	lipid binding	7.12E-03	255	38
GO:0045087	innate immune response	8.77E-03	276	39
GO:0051604	protein maturation	9.86E-03	84	36
GO:0050796	regulation of insulin secretion	1.32E-02	49	35
GO:0006805	xenobiotic metabolic process	1.41E-02	52	35
GO:0019058	viral life cycle	2.04E-02	39	30
GO:0000082	G1/S transition of mitotic cell cycle	2.08E-02	76	33
GO:0007399	nervous system development	2.13E-02	188	38
GO:0006024	glycosaminoglycan biosynthetic process	2.33E-02	24	23
GO:0007215	glutamate receptor signaling pathway	2.41E-02	11	26
GO:0034199	activation of protein kinase A activity	2.42E-02	11	20

GO:0006600	creatine metabolic process	2.45E-02	8	10
GO:0006913	nucleocytoplasmic transport	2.45E-02	146	37
GO:0007009	plasma membrane organization	2.49E-02	57	35
GO:0000718	nucleotide-excision repair, DNA damage removal	2.70E-02	12	17
GO:0032201	telomere maintenance via semi-conservative replication	2.86E-02	10	21
GO:0007158	neuron cell-cell adhesion	2.91E-02	13	21
GO:0031093	platelet alpha granule lumen	3.08E-02	17	25
GO:0042393	histone binding	3.23E-02	83	38
GO:0051437	positive regulation of ubiquitin-protein ligase activity involved in mitotic cell cycle	3.26E-02	26	30
GO:0003714	transcription corepressor activity	3.40E-02	116	39
GO:0030574	collagen catabolic process	3.41E-02	34	24
GO:0005070	SH3/SH2 adaptor activity	3.68E-02	30	30
GO:0030049	muscle filament sliding	3.77E-02	17	21
GO:0015939	pantothenate metabolic process	3.82E-02	8	17
GO:0042592	homeostatic process	3.97E-02	309	39
GO:0007603	phototransduction, visible light	4.10E-02	36	31
GO:0030148	sphingolipid biosynthetic process	4.55E-02	21	21
GO:0000096	sulfur amino acid metabolic process	4.78E-02	12	18

* p-value is FDR-adjusted.

Supplemental Figure S1. PCA Analysis. Cumulative variance explained by each principal component.

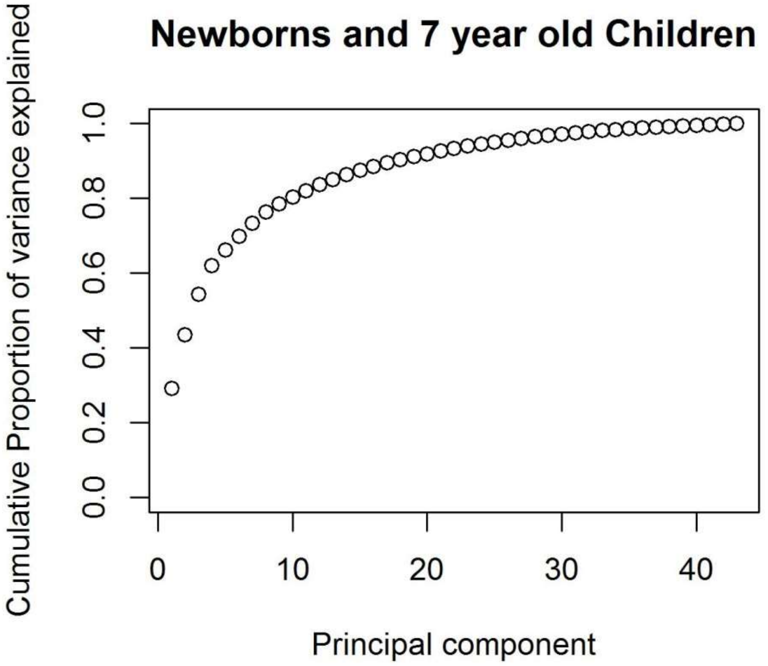

Supplement: Supplementary file 1 [file ijerph-16-00524-s001.pdf]
